# Progress in Medicine

**Published:** 1895-02-02

**Authors:** 


### Progress in Medicine.

### DISEASES OF CHILDREN.

(*Continued from page* 296.)

**The Treatment of Tapeworm.**—Dr. Descroizilles[21] advises the following procedures: (1) A preparatory diet for a few days of fish or vegetable soup, eggs, milk, and bread in moderation; (2) milk only for twenty-four hours before giving the following medicine; (3) an emulsion containing two drachms of the ethereal extract of male fern and seven grains of calomel, one-fourth part to be taken every ten minutes until the whole has been taken; and (4) an enema of six drachms of castor oil some hours later. This treatment proved successful in the case of a girl aged twelve years who had tapeworm. Dr. Leslie Ogilvie[22] employs a preparatory course of diet similar to the above, and in addition he administer salines and alkalies to clear out all excess of mucus from the intestines. A purgative having been given at night, on the following morning a drachm and a half of the liquid extract of male fern is administered in two doses, one at eight a.m. and the other at nine a.m., and then, whether the worm has been passed or not, a further dose of castor oil is given at eleven a.m., so as to wash out all remains of the male fern and prevent any toxic effects. He has never found any bad results follow from this treatment in children, whereas the administration of repeated daily doses of male fern is apt to produce nausea, faintness, and other symptoms of poisoning.

**Nasal Breathing in Nasal Obstruction.**—Mr. C. A. Parker[23] has made a series of observations as to the breathing during sleep of children who are suffering from nasal obstruction. He took a small piece of cotton wool, and, by holding this in front of the nose and then of the mouth, he was able to determine by which passage or passages the air was entering. Of 50 cases examined he found that in 82 per cent. the breathing was entirely nasal, and in only 2 per cent. was it entirely oral, while in the remaining 16 per cent.

the breathing was partly nasal and partly oral. He explains the restless sleep of patients suffering from this disorder by the fact that they make great efforts to get sufficient air through the nose, and failing in this they are awakened, draw a few full inspirations through the mouth, and fall asleep again, only to repeat the struggle in a short time. The writer then proceeds to discuss the various results which follow from nasal obstruction.

**Gavage or Forced Feeding.**—Dr. Emmet Holt[24] has found this very useful in the following cases: (1) Persistent vomiting due to habit, or exaggerated pharyngeal reflex; (2) acute diseases where, for any reason, children refuse all food, or struggle violently against everything that is offered them; (3) serious brain disease, when the patient cannot be fed by ordinary means. The apparatus employed consists of a funnel, eighteen inches of rubber tubing, a soft rubber catheter, and a few inches of glass tubing for connection. The child is placed on its back, and the head steadied by an assistant. The catheter, previously oiled, is then passed rapidly down the pharynx until nine or ten inches have been passed. Any gas present in the stomach is allowed an exit, and then the food is poured into the funnel and passes rapidly into the stomach. The food thus administered is seldom rejected.

**Enteric Fever in Infancy.**—Dr. W. B. Noyes[25] describes a number of cases of typhoid fever in infants, which had occurred in the course of an epidemic from a polluted milk supply. He thinks that this affection is not so rare amongst infants as is usually supposed, but that frequently it is not recognised. In the diagnosis he lays stress on the following points: A long-continued fever, which will not yield to quinine, and which the physical signs do not explain; gastro-intestinal disorder, with a tendency to constipation and putrid motions; an eruption of rose-coloured spots; symptoms of headache, such as rubbing the head, nose, and ears; a coated tongue with red edges, and later aphthous stomatitis; enlargement of the spleen, which, however, is often difficult to make out; and a moderate amount of tympanites and bronchitis. Cæcal tenderness and gurgling are of no value, being associated with so many disorders of infancy. The graver complications, such as hæmorrhage and perforation, are rare. Cerebral symptoms are frequently present, and may simulate those of meningitis very closely. In the treatment he finds sponging to be the best antipyretic, and to be safer than bathing. The best food is that which leaves no undigested masses in the stools. The medicinal treatment is symptomatic; a mineral acid, with bismuth as a gastro-intestinal sedative; bromides for nervous symptoms; champagne, ammonia, camphor, digitalis, and strychnine for cardiac weakness.

A case of typhoid fatal from perforation is recorded by Dr. S. R. Schofield.[26] The patient, a boy aged one year and nine months, was admitted into hospital three weeks after the onset of illness, as the gastro-intestinal symptoms had become rapidly worse. Vomiting, diarrhœa, and abdominal distension were present, but the spleen was not perceptibly enlarged, and no typhoid spots were visible. He died within a few days of admission. At the necropsy the spleen was found to be soft and slightly enlarged. A few flakes of lymph were scattered over the coils of the intestines. Adhesions had formed between a coil of intestine and the cæcum, and on separating them a cavity was opened into containing fæcal matter. This cavity communicated with the lower end of the ileum by a clean cut perforation in a Peyer's patch. A dozen other ulcers were present in the small intestine, and two in the cæcum, all being characteristically typhoid in appearance.

**The Insane Disorders of Childhood.**—Dr. J. Madison Taylor[27] considers that precocity in a child is frequently a sign of mental instability, and is apt to be followed by neuroses of various kinds. Excessive mental stimulation, rapid development of the emotions, heat and cold, fright, masturbation, and deprivation of food for long periods, are factors which play an important part in developing tendencies to latent disease in the growing brains of children. On the other hand, very much can be done by proper management and training in warding off the occurrence of permanent brain mischief. Mothers are often less successful with their own children than a stranger, from emotional and other considerations. Hysteria is often blended with organic mental disorders in childhood, and as there are not the same clinical phenomena of the former as one meets with in adult life, the physician must depend on his personal judgment, aided by any collateral evidence he can obtain. Moral imbeciles prove very hopeless cases, and at best a prolonged period of training must elapse before any symptoms of improvement can be looked for. In the management of hysterical and maniacal children, Dr. Taylor lays stress on the importance of obtaining the confidence and respect of the child by a persistently frank, quiet, and friendly demeanour. At the same time, obedience must be insisted on, and corporal punishment employed if necessary, for nothing is more disastrous to a moral imbecile than the belief that he can disobey with impunity or deceive with success.

[21] Jour. des Pratic, May 26th, 1894., and Therap. Gaz., Sept. 15th, 1894. [22] Lancet II., 1894, p. 255. [23] St. Barthol. Hosp. Rep., vol. xxix., p. 235. [24] N. Y. Med. Rec., Ap. 28th, 1894. [25] N. Y. Med. Rec., July 7th, 1894, p. 1. [26] B.M.J., Oct. 13th, 1894. [27] Arch. of Ped., Feb., 1894, and Int Med. Mag., May, 1894.

